# Postcardiotomy extracorporeal life support after isolated or valve-concomitant coronary artery bypass grafting: An observational multicenter study

**DOI:** 10.1016/j.xjon.2026.101875

**Published:** 2026-06-03

**Authors:** Silvia Mariani, Sacha Matteucci, Bas C.T. van Bussel, Anne-Kristin Schaefer, Diyar Saeed, Matteo Pozzi, Luca Botta, Nikolaos Kalampokas, Agne Jankuviene, Karl Bounader, Xiaotong Hou, Jeroen J.H. Bunge, Hergen Buscher, Leonardo Salazar, Bart Meyns, Michael A. Mazzeffi, Sandro Sponga, Vitaly Sorokin, Claudio Russo, Michele Di Mauro, Pranya Sakiyalak, Antonio Fiore, Daniele Camboni, Giuseppe Maria Raffa, Rodrigo Diaz, I-wen Wang, Jae-Seung Jung, Jan Belohlavek, Vin Pellegrino, Giacomo Bianchi, Matteo Pettinari, Alessandro Barbone, José P. Garcia, Kiran Shekar, Glenn Whitman, Francesco Formica, Roberto Lorusso

**Affiliations:** a Cardiovascular Research Institute Maastricht, Maastricht University, Maastricht, The Netherlands; b Cardiac Surgery Unit, Cardio-Thoracic and Vascular Department, Fondazione IRCCS San Gerardo dei Tintori, Monza, Italy; c Cardiovascular Department, Azienda Ospedaliero Universitaria delle Marche, Ospedali Riuniti 'Umberto I – G.M. Lancisi – G. Salesi' Università Politecnica delle Marche, Ancona, Italy; d Department of Intensive Care Medicine, Maastricht University Medical Center, Maastricht, The Netherlands; e Care and Public Health Research Institute, Maastricht University, Maastricht, The Netherlands; f Division of Cardiac Surgery, Medical University of Vienna, Vienna, Austria; g Department of Cardiovascular Surgery, Heart Center Niederrhein, Health and Medical University Krefeld, Krefeld, Germany; h Department of Cardiac Surgery, Louis Pradel Cardiologic Hospital, Lyon, France; i Division of Cardiac Surgery, IRCCS Azienda Ospedaliero-Universitaria di Bologna, Bologna, Italy; j Department of Cardiac Surgery, Medical Faculty, Heinrich Heine University, Duesseldorf, Germany; k II Department of Anesthesiology, Centre of Anesthesia, Intensive Care and Pain Management, Vilnius University Hospital Santariskiu Klinikos, Vilnius, Lithuania; l Division of Cardiothoracic and Vascular Surgery, Pontchaillou University Hospital, Rennes, France; m Center for Cardiac Intensive Care, Beijing Institute of Heart, Lung, and Blood Vessels Diseases, Beijing Anzhen Hospital, Capital Medical University, Beijing, China; n Departmens of Cardiology, Erasmus MC, Rotterdam, The Netherlands; o Department of Intensive Care Adults, Erasmus MC, Rotterdam, The Netherlands; p Department of Intensive Care Medicine, Center of Applied Medical Research, St Vincent's Hospital, Darlinghurst, New South Wales, Australia; q Department of Cardiology, Fundación Cardiovascular de Colombia, Bucaramanga, Colombia; r Department of Cardiac Surgery, University Hospitals Leuven and Department of Cardiovascular Sciences, University of Leuven, Leuven, Belgium; s Departments of Medicine and Surgery, University of Maryland, Baltimore, Md; t Division of Cardiac Surgery, Cardiothoracic Department, University Hospital of Udine, Udine, Italy; u Cardiothoracic Intensive Care Unit, Department of Cardiac, Thoracic and Vascular Surgery, National University Hospital, Singapore; v Cardiac Surgery Unit, Cardiac Thoracic and Vascular Department, Niguarda Hospital, Milan, Italy; w Division of Cardiac Surgery, Department of Medical and Surgical Sciences, University of Foggia, Foggia, Italy; x Division of Cardiovascular and Thoracic Surgery, Department of Surgery, Faculty of Medicine Siriraj Hospital, Mahidol University, Bangkok, Thailand; y Department of Cardio-Thoracic Surgery, University Hospital Henri-Mondor, Créteil, Paris, France; z Department of Cardiothoracic Surgery, University Medical Center Regensburg, Regensburg, Germany; aa Cardiac Surgery Unit, Department of Precision Medicine in Medical Surgical and Critical Area (Me.Pre.C.C.), University of Palermo, Palermo, Italy; bb Department for the Treatment and Study of Cardiothoracic Diseases and Cardiothoracic Transplantation, IRCCS-ISMETT (Istituto Mediterraneo per i Trapianti e Terapie ad Alta Specializzazione), Palermo, Italy; cc ECLS Unit, Departamento de Anestesia, Clínica Las Condes, Las Condes, Santiago, Chile; dd Division of Cardiac Surgery, Memorial Healthcare System, Hollywood, Fla; ee Department of Thoracic and Cardiovascular Surgery, Korea University Anam Hospital, Seoul, South Korea; ff 2nd Department of Internal Medicine, Cardiovascular Medicine General Teaching Hospital and 1st Faculty of Medicine, Charles University in Prague, Prague, Czech Republic; gg Intensive Care Unit, The Alfred Hospital, Monash University, Melbourne, Victoria, Australia; hh Department of Adult Cardiac Surgery, Ospedale del Cuore, Fondazione Toscana “G. Monasterio”, Massa, Italy; ii Department of Cardiovascular Surgery, Ziekenhuis Oost-Limburg, Genk, Belgium; jj Cardiac Surgery Unit, Department of Cardiac Surgery, IRCCS Humanitas Research Hospital, Rozzano, MI, Italy; kk IU Health Advanced Heart & Lung Care, Indiana University Methodist Hospital, Indianapolis, Ind; ll Adult Intensive Care Services, The Prince Charles Hospital, Brisbane, Queensland, Australia; mm Cardiac Intensive Care Unit, Department of Cardiac Surgery, Johns Hopkins University, School of Medicine, Baltimore, Md; nn Department of Experimental Medicine, University of Salento, Lecce, Italy

**Keywords:** extracorporeal life support, postcardiotomy cardiogenic shock, valve surgery, coronary-artery-bypass

## Abstract

**Objectives:**

Data on outcomes of patients undergoing isolated coronary artery bypass grafting (isolated CABG) versus CABG and concomitant left heart valve (LHV-CABG) surgery are conflicting, especially in extracorporeal life support (ECLS) settings. We compared characteristics, in-hospital outcomes, and overall survival between patients undergoing isolated-CABG and concomitant LHV-CABG requiring postcardiotomy ECLS from a large multicenter study.

**Methods:**

This retrospective, multicenter (34 centers), observational study included adults requiring postcardiotomy ECLS between 2000 and 2020. Clinical characteristics and outcomes were compared between patients who underwent isolated CABG with those who underwent LHV-CABG. Association between type of surgery and in-hospital survival was investigated through mixed-Cox proportional hazards models.

**Results:**

This study included 639 patients comprising 58.8% (n = 376) isolated CABG and 41.1% (n = 263) LHV-CABG, including 46.7% (n = 123) aortic, 38.8% (n = 102) mitral, and 14.5% (n = 38) combined aortic-mitral valve procedures. The LHV-CABG patients were older (*P* = .001), more frequently experienced preoperative pulmonary artery hypertension (*P* < .001), and 6.5% (n = 17) had active endocarditis. They required longer cardiopulmonary bypass times (*P* < .001) and cardiac surgery reoperations (*P* = .002). In-hospital mortality was 54.8% (n = 206) and 63.1% (n = 166) in the isolated CABG and LHV-CABG groups (*P* = .036), respectively. Crude hazard ratio for in-hospital mortality in LHV-CABG was 1.28 (95% CI, 1.03-1.58, *P* = .023) and did not change after adjustments. The 5-year postdischarge survival probabilities were 71.1% (95% CI, 61.0-82.2) and 69.3% (95% CI, 57.7-83.2; *P* = .210) for isolated CABG and LHV-CABG groups, respectively.

**Conclusions:**

LHV-CABG surgery, compared with isolated CABG, was associated with higher in-hospital mortality in patients requiring ECLS, whereas no midterm postdischarge survival differences could be detected. Early identification of patients in need for ECLS following LHV-CABG may improve outcomes.

**Figure:**
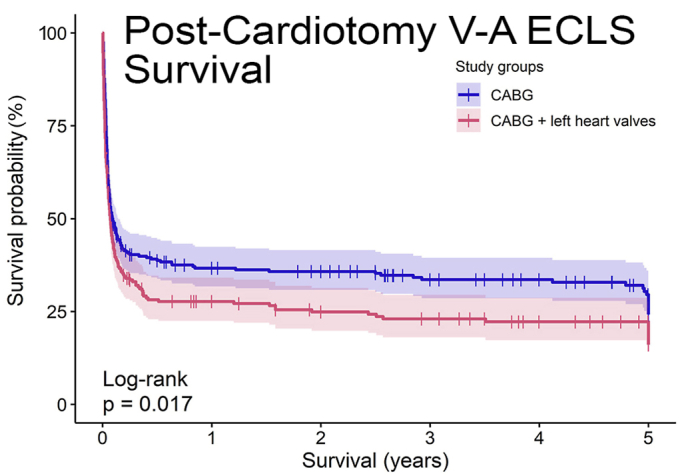
Venoarterial ECLS after isolated CABG or concomitant left heart valve CABG.

**Central Message**

ECLS after coronary artery bypass grafting with valve surgery shows higher in-hospital mortality, but similar 5-year postdischarge survival compared with isolated coronary artery bypass grafting.

**Perspective**

Postcardiotomy-ECLS following concomitant LHV-CABG shows higher in-hospital mortality than after isolated CABG, despite similar complications and 5-year postdischarge survival. This finding underscores the need for targeted risk stratification and timely ECLS initiation in patients with high-risk LHV-CABG.

Over the past 2 decades, advances in technology and increased patient complexity have transformed cardiac surgery. Whereas surgeons once treated younger patients with isolated diseases, they now routinely manage older individuals, with multiple cardiac pathologies and comorbidities. In addition, many procedures, such as isolated coronary revascularization or isolated aortic valve replacement, are nowadays replaced by transcatheter interventions.^1^^,^^2^ The number of isolated coronary artery bypass grafting (isolated CABG) procedures has decreased by 34% from 2002 to 2016 while there has been a consistent rise in the number of patients undergoing valve surgery, with an increase by 71% from 2002 to 2016 in the United Kingdom.^3^ These numbers indicate a growing prevalence of combined procedures. Despite these trends, data on early and late outcomes of patients undergoing isolated CABG versus CABG and concomitant left heart valve surgery (LHV-CABG) are conflicting.^4^, ^5^, ^6^ A percentage of these patients might require perioperative extracorporeal life support (ECLS).^7^^,^^8^ Nevertheless, data on early outcomes are even more contradictory in this population due to the sparse literature and the uncertain results.^9^ Only few large studies have explored specific predictors of survival in this population.^10^^,^^11^ Postcardiotomy ECLS (PC-ECLS) may offer a bridge to recovery or further interventions, but survival remains around 40% or lower in patients undergoing complex procedures.^10^^,^^12^^,^^13^ Thus, management, mortality, and long-term outcomes remain unexplored topics in patients receiving ECLS post-CABG with or without repaired or replaced valves,^14^ leaving clinicians with open questions and doubts about their patients’ outcomes.

This study aimed to compare characteristics and outcomes of patients receiving PC-ECLS following isolated CABG versus concomitant LHV-CABG surgery. We hypothesized that PC-ECLS after isolated CABG is associated with lower in-hospital and postdischarge mortality and complications rates compared with LHV-CABG.

## Methods

### Study Design

The current study is a secondary post hoc analysis of the Post-Cardiotomy Extracorporeal Life Support (PELS) investigation (ClinicalTrials.gov identifier: NCT03857217), an international, multi-center, retrospective observational study on PC-ECLS collecting data from 34 centers and 16 countries.^11^ The current study was conducted in accordance with the Declaration of Helsinki. Ethical approval was acquired in all centers based on the coordinating center approval (METC-2018–0788; December 2018). Informed consent was waived due to the observational approach, the emergency of the performed procedure, and the de-identification of shared data. The study is reported following the Strengthening the Reporting of Observational Studies in Epidemiology statement (Appendix E2).^15^

### Patient Population

**Figure E1:**
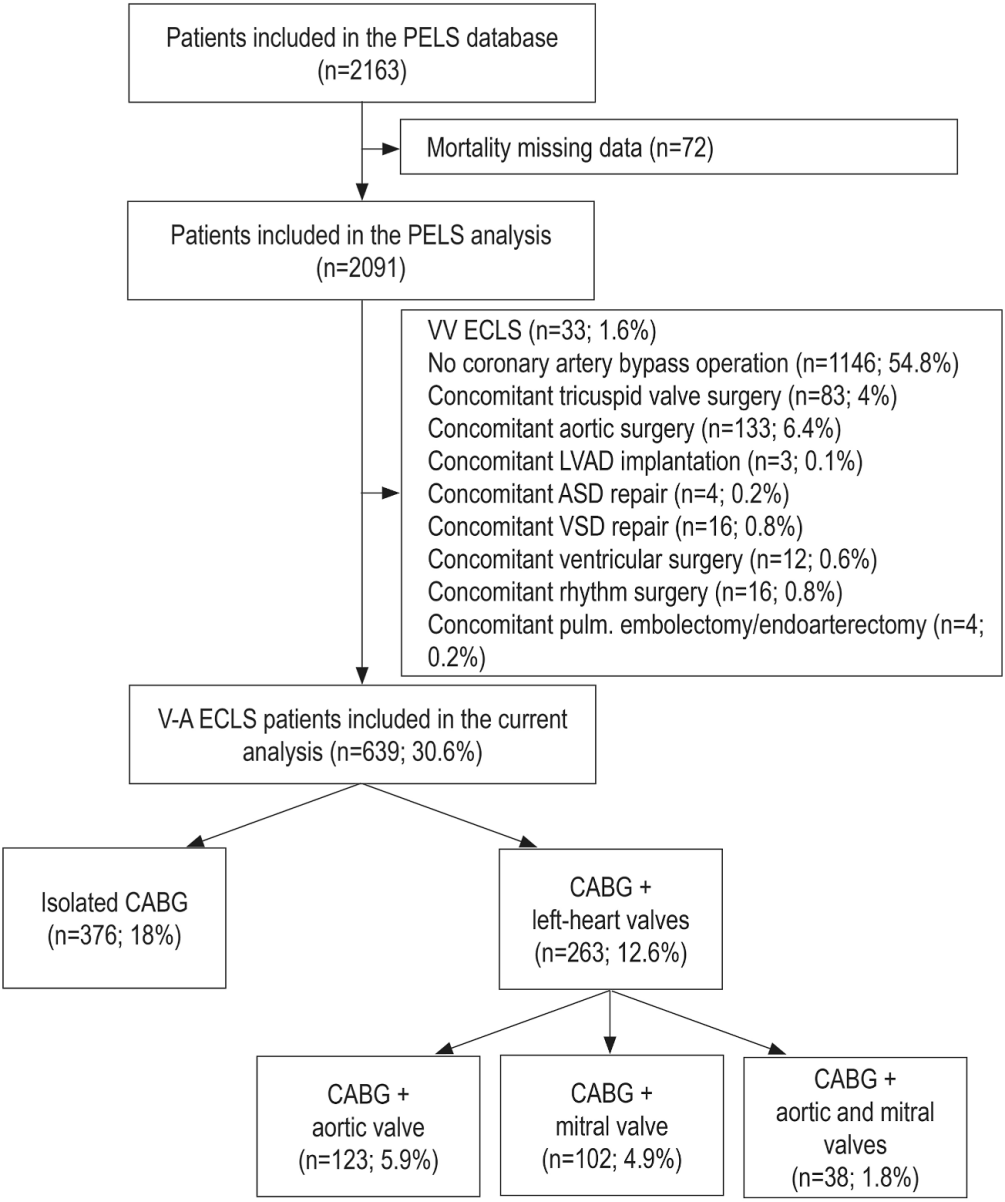
Patients flow chart. Percentages are calculated on the overall number of patients included in the analysis. *ASD*, Atrial septal defect; *CABG*, coronary artery bypass graft; *ECLS*, extracorporeal life support; *LVAD*, left ventricular assist device; *PELS*, post-cardiotomy extracorporeal life support study; *VSD*, ventricular septal defect; *VA*, venoarterial; *VV*, venovenous.

PELS included adults (ages 18 years and older) requiring central, peripheral, or combined PC-ECLS between January 2000 and December 2020. Exclusion criteria encompassed ECLS after discharge or before surgery, ECLS after noncardiac operations, and ECLS implantation not related to cardiac surgery hospitalization. For the current analysis, we considered only patients who underwent venoarterial ECLS after isolated CABG or concomitant LHV-CABG and excluded patients who underwent venovenous ECLS, CABG associated with any procedure other than mitral and/or aortic valve surgery, or with missing data on primary outcome (Figure E1).

### Data Collection and Outcomes

Data on patients’ characteristics and outcomes were collected in a dedicated electronic database (data.castoredc.com) according to the predefined protocol and variable definitions (Appendix E3). Follow-up data were collected through review of medical records or contact with patients at the discretion of the treating center. The primary outcome of interest was all-cause in-hospital mortality. Secondary outcomes included in-hospital complications (eg, stroke, bleeding, and sepsis) and postdischarge survival.

### Statistical Analysis

**Figure E2:**
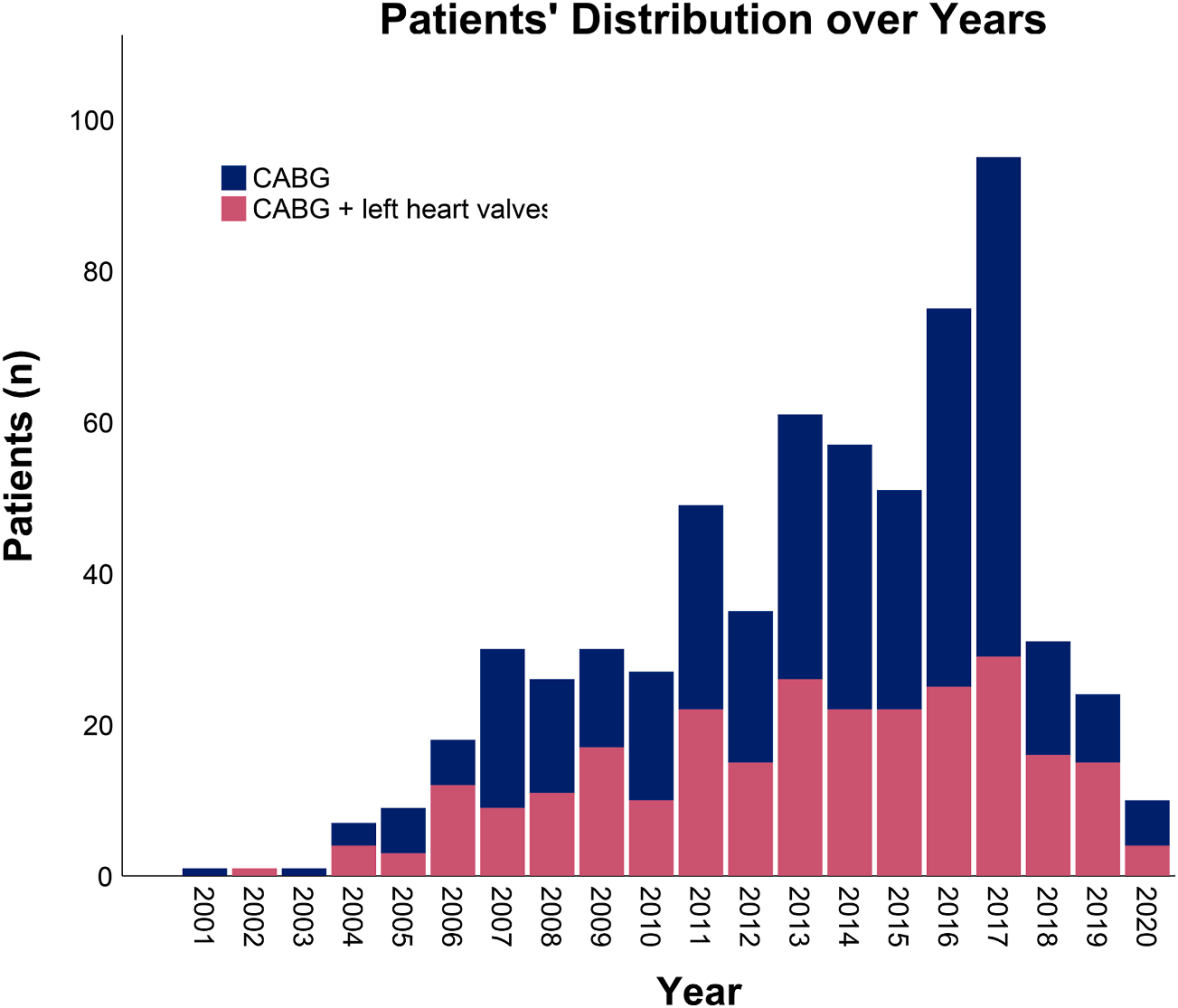
Stacked bar charts representing the distribution of patients over the study period. *CABG*, Coronary artery bypass graft.

Data were merged and analyzed using SPSS 26.0 (IBM) and R 4.1.2 (R Foundation for Statistical Computing) (see Appendix E3). The full cohort was categorized into 2 study groups (isolated CABG vs LHV-CABG). Missing data were analyzed and reported (Table E1). Descriptive statistics were conducted on available data only. Normality was investigated. Demographic and clinical variables are expressed as numbers (valid percent on available data, excluding missing values) for categorical variables and median (interquartile range [IQR]) for continuous variables. Categorical variables were compared with Pearson χ^2^, Fisher exact test, or Fisher-Freeman-Halton exact test. Continuous variables were analyzed with Mann-Whitney *U* test. Overall survival was investigated with Kaplan-Meier method and comparisons were performed with log rank test. Curves were truncated when the number of patients at risk dropped below 10% of the initial sample. The association between type of surgery and in-hospital mortality was investigated with mixed-effects Cox proportional hazards regression models. We first estimated a crude model, and subsequently adjusted for preoperative, intraoperative, ECLS variables, and complications. All models contained both fixed and random effects to account for dependency of observations due to clustering in centers and in years. The models were developed on 5 datasets after imputation of variables with <20% missing data. We report measures of association as hazard ratios (HRs) with their 95% CIs and *P* values. Based on the possible variations in ECLS management and prosthetic valve availability over the study period (Figure E2), a sensitivity analysis excluding patients who received PC-ECLS between 2000 and 2010 was performed. All data were merged from deidentified files into SPSS 26.0 software and R 4.1.2 for statistical analysis.

## Results

### Preoperative, Surgical, and ECLS Characteristics

Out of 2091 patients included in the PELS study, 639 patients were included in the current analysis: 376 (58.8%) underwent isolated CABG and 263 (41.2%) LHV-CABG. The latter group included 123 (46.7%) patients undergoing aortic valve surgery, 102 (38.8%) undergoing mitral valve procedures, and 38 (14.5%) who received combined aortic and mitral valves surgery (Figure E1). The LHV-CABG patients were older (isolated CABG, age 66.1 years; IQR, 58-72.5 years; LHV-CABG, age 70 years; IQR, 61.9-74 years; *P* = .001), with a higher female prevalence than the isolated CABG group (isolated CABG, n = 118 [31.4%]; LHV-CABG, n = 107 [40.7%]; *P* = .020), and more frequently with preoperative atrial fibrillation (*P* = .03), pulmonary artery hypertension (*P* < .0001), right ventricular failure (*P* = .03), and septic shock (*P* = .019, Table 1). Although LHV-CABG recipients were less frequently operated on in an emergency situation (*P* < .001) or in cardiogenic shock (*P* = .002), they had a higher rate of reinterventions (*P* = .004) and required longer cardiopulmonary bypass (CPB) times (isolated CABG, 122 minutes; IQR, 100-191 minutes; LHV-CABG, 220 minutes; IQR, 160-305 minutes; *P* = .0001) (Table 2). The main indication for ECLS initiation was failure to wean from CPB (isolated CABG, n = 105 [28.3%]; LHV-CABG: n = 123 [48.2%]; *P* < .001) (Table 3). The LHV-CABG group showed a higher rate of intraoperative ECLS initiation (isolated CABG, n = 188 [50%]; LHV-CABG, n = 171 [65%]), whilst postoperative ECLS implantation was more frequent in the isolated CABG group (isolated CABG, n = 188 [50%]; LHV-CABG, n = 92 [35%]; *P* < .001). The use of an intra-aortic balloon pump was more frequent in the isolated CABG group (isolated CABG, n = 204 [55%]; LHV-CABG, n = 101 [38.5%]; *P* < .001). Median ECLS support duration was 120 hours (IQR, 64.5-197 minutes).

**Table 1:** Baseline characteristics of the overall population

Variables	Overall population (N = 639)	Isolated CABG (n = 376)	LHV-CABG (n = 263)	*P* value
Case distribution over years				.343
2000-2010	150 (23.5)	83 (22.1)	67 (25.5)	
2011-2020	489 (76.5)	293 (77.9)	196 (74.5)	
Age (y)	67.9 (59.0-73.1)	66.1 (58.0-72.5)	70.0 (61.9-74.0)	.001
Sex				.020
Female	225 (35.2)	118 (31.4)	107 (40.7)	
Male	414 (64.8)	258 (68.6)	156 (59.3)	
Body mass index	26.6 (24 - 30.1)	26.8 (24.2-29.7)	26.6 (23.9-30.8)	.610
Comorbidities				
Hypertension	488 (77.8)	284 (76.8)	204 (79.4)	.430
Dialysis	56 (9.1)	31 (8.5)	25 (10.0)	.548
Previous AMI	314 (49.1)	218 (58.0)	96 (36.5)	<.001
AMI (within last 30 d)	153 (24.4)	108 (29.2)	45 (17.5)	.001
Smoking	177 (31.8)	116 (34.19)	61 (28.1)	.160
Previous stroke	76 (11.9)	38 (10.1)	38 (14.4)	.100
Atrial fibrillation	109 (17.1)	54 (14.4)	55 (21.0)	.030
Diabetes mellitus	243 (38.0)	149 (39.6)	94 (35.7)	.320
Implanted pacemaker	32 (5.4)	13 (3.7)	19 (7.9)	.027
Implantable cardioverter defibrillator	11 (1.8)	2 (0.6)	9 (3.8)	.006
Previous percutaneous coronary intervention	146 (23.0)	98 (26.2)	48 (18.4)	.022
Chronic obstructive pulmonary disease	74 (12.1)	46 (12.6)	28 (11.2)	.617
Pulmonary artery hypertension (>50 mm Hg)	91 (14.3)	36 (9.6)	55 (21.0)	<.001
Previous cardiac surgery	67 (10.5)	28 (7.4)	39 (14.8)	.004
Left ventricular ejection fraction (%)	40 (30-55)	40 (29-55)	45 (32-60)	.002
EuroSCORE II	6.6 (2.7-18.7)	5.3 (2.3-16.6)	7.9 (3.3-22.3)	.003
Preoperative condition				
NYHA class				.193
Class I	39 (6.4)	27 (7.6)	12 (4.7)	
Class II	134 (22)	75 (21.1)	59 (23.2)	
Class III	250 (41.1)	137 (38.6)	113 (44.5)	
Class IV	186 (30.5)	116 (32.7)	70 (27.6)	
Cardiogenic shock	168 (26.8)	115 (31.3)	53 (20.3)	.002
Intubation	85 (13.3)	53 (14.1)	32 (12.2)	.554
Cardiac arrest	94 (14.9)	57 (15.2)	37 (14.5)	.909
Septic shock	12 (1.9)	2 (0.8)	9 (3.5)	.019
Vasopressors	121 (19.1)	79 (21.2)	42 (16.2)	.124
Right ventricular failure	23 (4.1)	9 (2.6)	14 (6.4)	.031
Emergency surgery	208 (32.5)	148 (39.7)	60 (23.3)	<.001
Urgent surgery	116 (18.4)	64 (17.2)	52 (20.2)	.348
Pre-operative creatinine (μmol/L)	97.3 (79.6-130)	97.2 (77.8-123.7)	103.4 (83-140)	.020
Active endocarditis	17 (2.7)	0 (0)	17 (6.5)	N/A

Data are reported as n (% as valid percentage excluding missing values) or median (interquartile range). *CABG*, Coronary artery bypass graft; *LHV*, left heart valve; *AMI*, acute myocardial infarction; *EuroSCORE II*, European System for Cardiac Operative Risk Evaluation II; *NYHA*, New York Heart Association; *N/A*, not applicable.

**Table 2:** Procedural characteristics

Variables	Overall population (N = 639)	Isolated-CABG (n = 376)	LHV-CABG (n = 263)	*P* value
Arterial conduits				.051
No arterial grafts	135 (26.8)	77 (24.3)	58 (31.0)	
1 arterial graft	283 (56.2)	177 (55.8)	106 (56.7)	
2 arterial grafts	86 (17.1)	63 (19.9)	23 (12.3)	
Aortic valve surgery			161 (61.2)	N/A
Aortic valve repair			31 (23.5)	
Biological prosthesis			83 (62.9)	
Mechanical prosthesis			18 (13.6)	
Mitral valve surgery			140 (53.2)	N/A
Mitral valve repair			62 (48.1)	
Biological prosthesis			40 (31.0)	
Mechanical prosthesis			27 (20.9)	
Cardiopulmonary bypass time (min)	169 (114-242)	132 (100-191)	220 (160-305)	.001
Crossclamp time (min)	80 (48-121)	55 (18-81)	116 (88-151)	.001
Intraoperative transfusions of any blood components	239 (94.5)	150 (93.8)	89 (95.7)	.582

Data are reported as n (% as valid percentage excluding missing values) or median (interquartile range). *CABG*, Coronary artery bypass graft; *LHV*, left heart valve; *N/A*, not applicable.

**Table 3:** Details on extracorporeal life support (ECLS)

Variables	Overall population (n = 639)	Isolated CABG (n = 376)	LHV-CABG (n = 263)	*P* value
ECLS indication				<.001
Failure to wean	228 (36.4)	105 (28.3)	123 (48.2)	
Acute pulmonary embolism	0 (0)	0 (0)	0 (0)	
Arrhythmia	25 (4.0)	22 (5.9)	3 (1.2)	
Cardiac arrest	77 (12.3)	56 (15.1)	21 (8.2)	
Cardiogenic shock	204 (32.6)	144 (38.8)	60 (23.5)	
Pulmonary hemorrhage	2 (0.3)	0 (0)	2 (0.8)	
Right ventricular failure	36 (5.8)	18 (4.9)	18 (7.1)	
Respiratory failure	20 (3.2)	9 (2.4)	11 (4.3)	
Biventricular failure	25 (4.0)	14 (3.8)	11 (4.3)	
Other	9 (1.4)	3 (0.8)	6 (2.4)	
ECLS cannulation site				.252
Only central	112 (17.5)	58 (15.4)	54 (20.5)	
Only peripheral	278 (43.5)	174 (46.3)	104 (39.5)	
Mixed/switch	229 (35.8)	133 (35.4)	96 (36.5)	
Unknown	20 (3.1)	11 (2.9)	9 (3.4)	
Distal perfusion cannula	238 (64.4)	153 (66.5)	85 (63.4)	.569
ECLS implant timing				<.001
Intraoperative	359 (56.2)	188 (50.0)	171 (65.0)	
Postoperative	280 (43.8)	188 (50.0)	92 (35.0)	
Chest status				.926
Closed	271 (55.6)	163 (55.8)	108 (55.4)	
Open	216 (44.4)	129 (44.2)	87 (44.6)	
Blood loss first 24 h (mL)	810 (450-1590)	700 (400-1500)	900 (500-1778)	.015
Postoperative PRBCs (n)	13 (5-26)	11 (5-26)	14 (5-26)	.697
IABP	305 (48.2)	204 (55)	101 (38.5)	<.001
IABP implant timing				.595
Preoperative	109 (35.7)	75 (36.8)	34 (33.7)	
Intraoperative	196 (64.3)	129 (63.2)	67 (66.3)	
Left ventricular unloading	216 (41.7)	143 (48.3)	73 (32.9)	<.001
ECLS duration (h)	120.0 (64.5-197.0)	120.0 (64.8- 214.0)	117.9 (62.3-192.0)	.267

Values are reported as n (% as valid percentage excluding missing values) or median (interquartile range). *CABG*, Coronary artery bypass graft; *LHV*, left heart valve; *PRBCs*, packed red blood cells; *IABP*, intra aortic balloon pump.

### Outcomes

**Figure 1:**
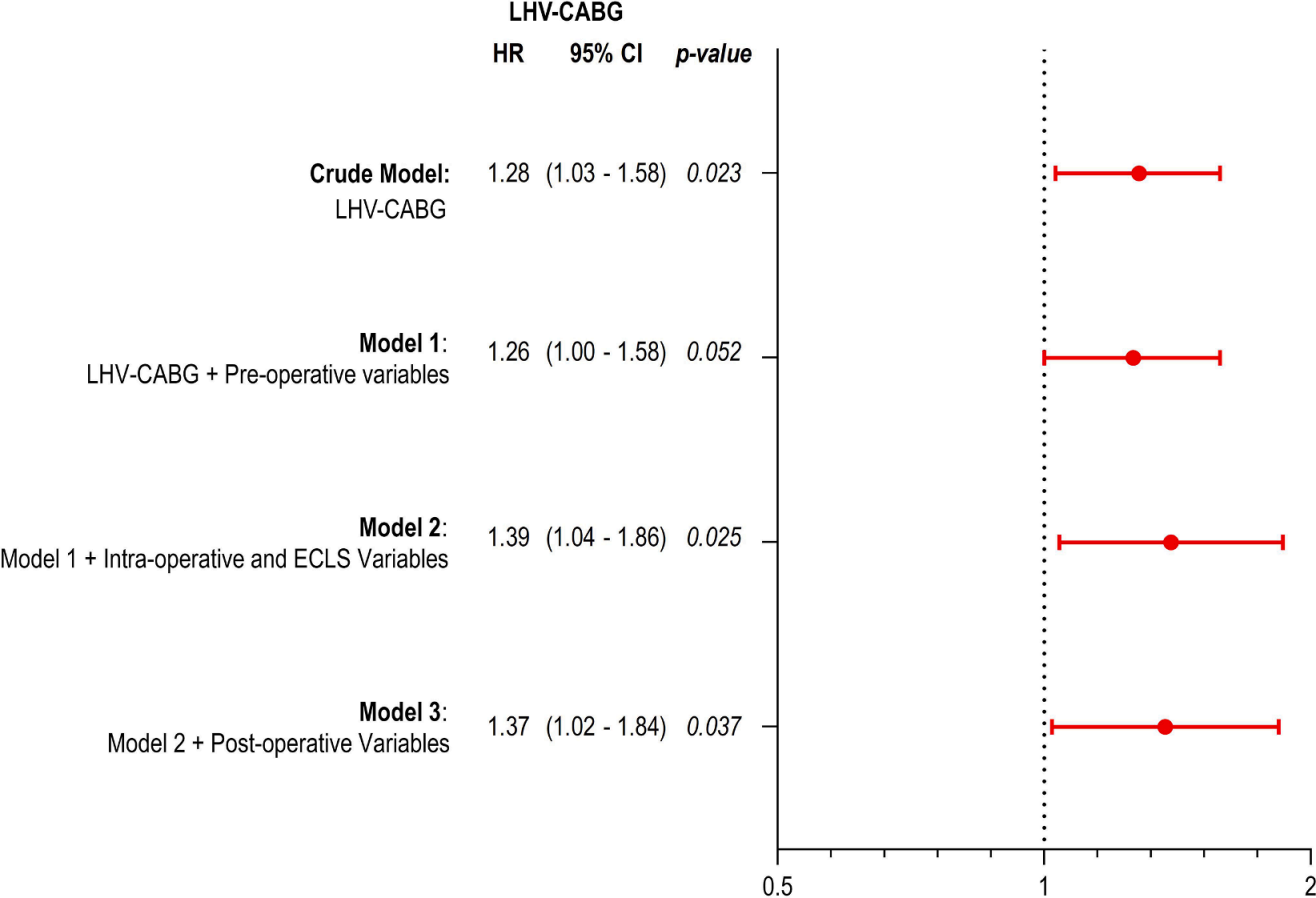
Hazard ratios for in-hospital mortality. In a mixed-effects Cox model with random center and year effect, left heart valve coronary artery bypass graft (*LHV-CABG*) showed a significantly higher hazard ratio for in-hospital mortality compared with isolated-CABG (crude model). Adjustment for demographic and preoperative characteristics (Model 2), intraoperative and extracorporeal life support (*ECLS*) variables (Model 3), and postoperative complications (Model 4) did not change the results.

**Figure 2:**
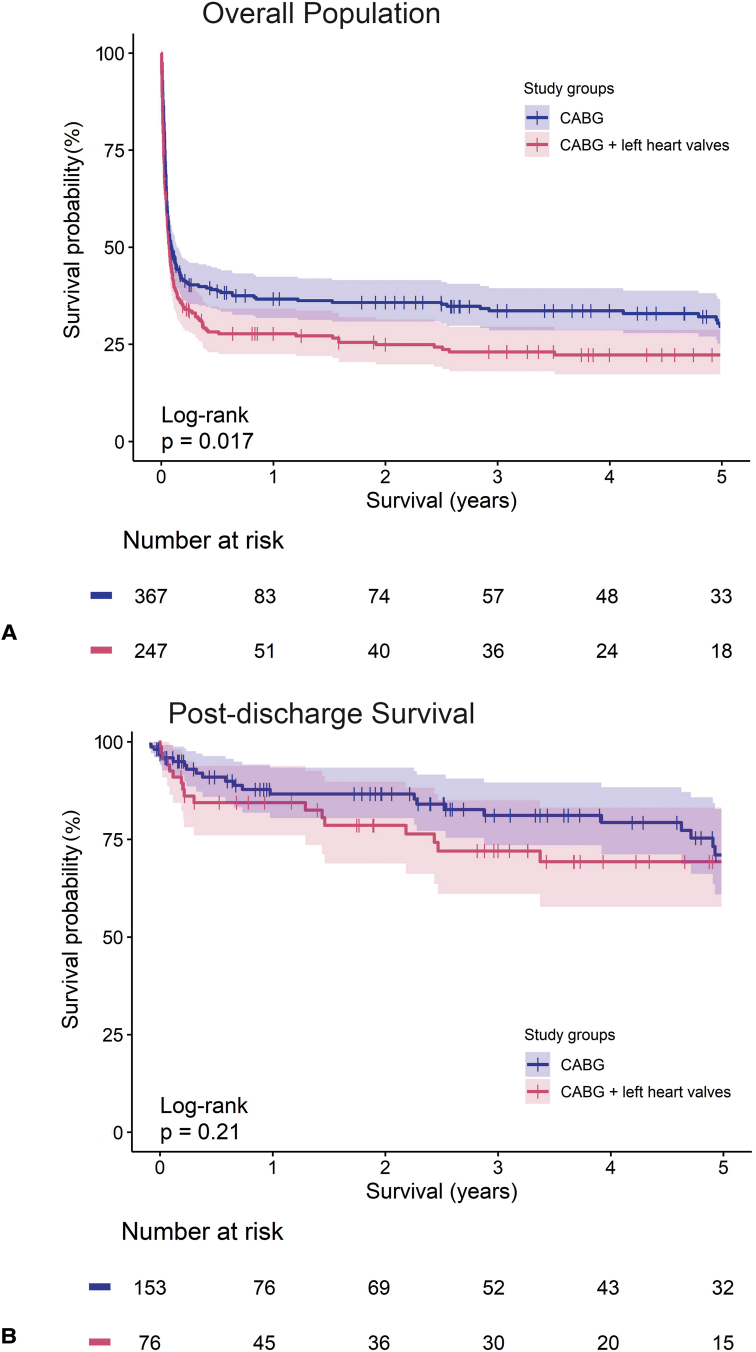
A, Kaplan-Meier survival curves with 95% CI representing 5-year survival in the overall population. B, Kaplan-Meier survival curves with 95% CI representing 5-year postdischarge survival. *CABG*, Coronary artery bypass graft.

**Figure E3:**
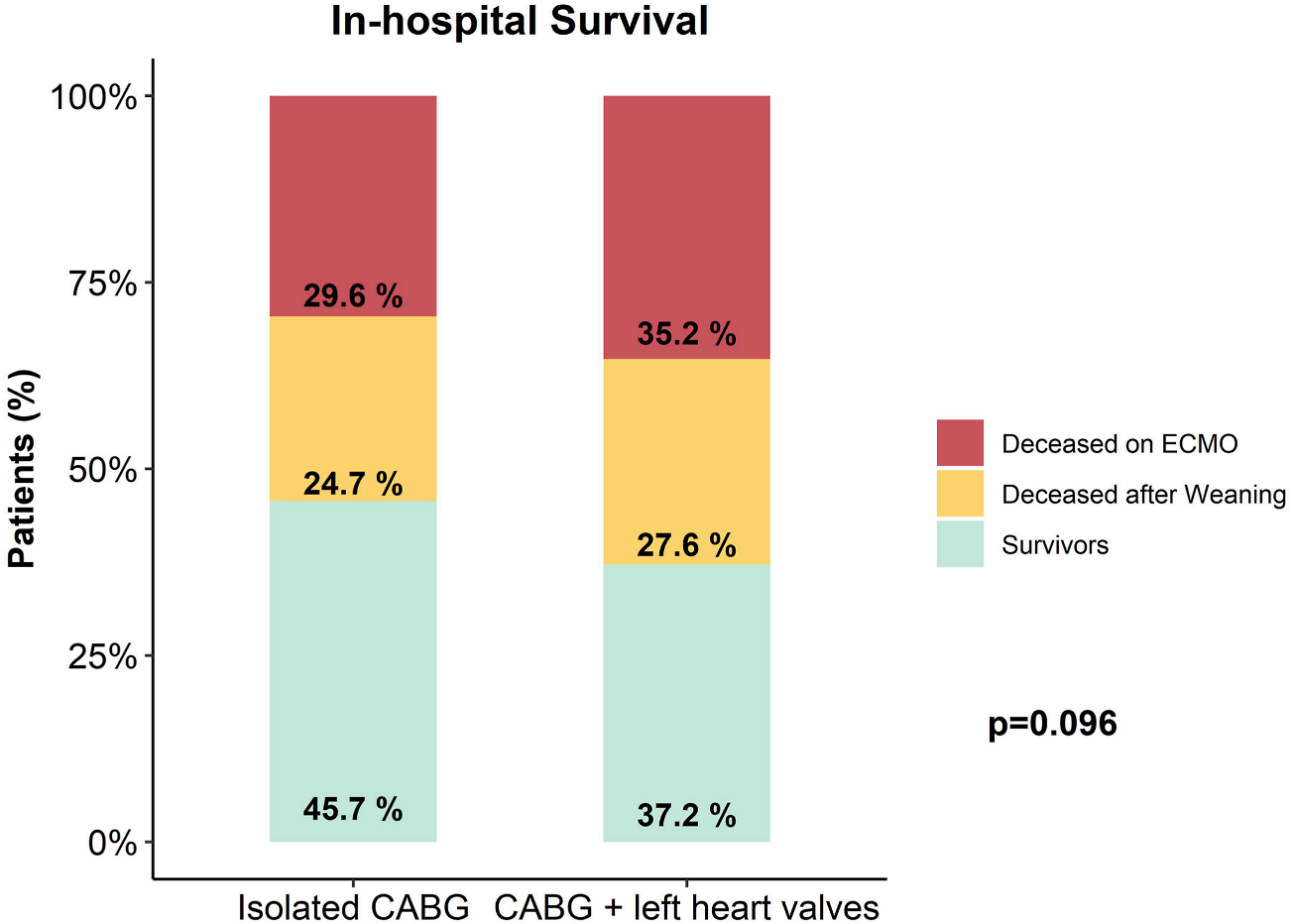
Stacked bar charts representing the distribution of patients based on in-hospital survival or death timing. *CABG*, Coronary artery bypass graft; *ECLS*, extracorporeal life support.

Mechanical ventilation, intensive care unit, and hospitalization times were comparable between groups (Table 4). Despite a higher blood loss during the first 24 hours (isolated CABG, 700 mL; IQR, 400-1500 mL; LHV-CABG, 900 mL; IQR, 500-1778 mL; *P* = .015), re-exploration for bleeding and postoperative transfusion rates were comparable between groups (Table 3). Cardiac surgery reoperations (*P* = .002) and postoperative pacemaker implantation (*P* = .017) were more frequent in the LHV-CABG group. In-hospital mortality was higher for LHV-CABG patients (isolated CABG, n = 206 [54.8%]; LHV-CABG, n = 166 [63.1%]; *P* = .036) regardless the death timing (Figure E3). In a mixed-effects Cox model with random center and year effects, LHV-CABG surgery showed a higher hazard for in-hospital mortality (HR, 1.28; 95% CI, 1.03-1.58; *P* = .023) (Figure 1 and Table E2) compared with isolated CABG, also after model adjustment (HR, 1.37; 95% CI, 1.02-1.84; *P* = .037). Kaplan-Meier curves confirmed an overall lower survival probability for LHV-CABG patients (*P* = .017) (Figure 2, *A*). When considering only hospital survivors with available data on follow-up, the 5-year survival probabilities were 71.1% (95% CI, 61.0%-82.2%) and 69.3% (95% CI, 57.7%-83.2%; *P* = .210) (Figure 2, *B*) for isolated CABG and LHV-CABG groups, respectively. The sensitivity analysis after exclusion of patients operated on between 2000 and 2010 (n = 489) showed minor discrepancies (Table E3, Table E4, Table E5, Table E6) despite a rise in the use of biological valves (Table E7).

**Table 4:** Postoperative outcomes

Variables	Overall population (N = 639)	Isolated-CABG (n = 376)	LHV-CABG (n = 263)	*P* value
Mechanical ventilation time (h)	10 (4-17)	10 (4-15.5)	10 (5-18)	.412
Intensive care unit stay (d)	14 (7-25)	14 (7-24)	15 (6-29)	.649
Hospital stay (d)	20 (10-35)	20 (11-35)	21 (8-37)	.6
Postoperative complications				
Any bleeding	343 (54.4)	195 (52.6)	148 (56.9)	.292
Bleeding requiring rethoracotomy	222 (36.9)	122 (34.4)	100 (40.5)	.144
Cannulation site bleeding	82 (13.0)	46 (12.4)	36 (13.9)	.631
Diffuse no surgical related bleeding	14 (23.7)	87 (24.4)	54 (22.8)	.694
Neurological complications	409 (83.5)	253 (81.4)	156 (87.2)	.102
Intracerebral bleeding	15 (2.5)	9 (2.5)	6 (2.4)	1.000
Brain edema	25 (4.0)	16 (4.4)	9 (3.5)	.681
Seizure	18 (2.9)	9 (2.5)	9 (3.5)	.472
Cerebral hemorrhage	20 (3.3)	11 (3.0)	9 (3.6)	.818
Stroke	69 (10.9)	43 (11.5)	26 (10.0)	.605
Arrhythmia	218 (36.5)	138 (38.7)	80 (33.3)	.194
Leg ischemia	70 (11.5)	42 (11.6)	28 (11.4)	1.000
Cardiac arrest	111 (18.6)	61 (17.0)	50 (20.9)	.239
Pacemaker implant	12 (2.0)	3 (0.8)	9 (3.8)	.017
Bowel ischemia	36 (5.6)	21 (5.9)	15 (6.3)	.862
Right ventricular failure	94 (16.1)	48 (13.6)	46 (19.7)	.051
Acute kidney injury	324 (54.6)	185 (52.3)	139 (58.2)	.179
Pneumonia	122 (20.9)	76 (21.7)	46 (19.8)	.605
Septic shock	76 (13.0)	45 (12.8)	31 (13.4)	.9
Distributive shock syndrome	43 (7.4)	26 (7.4)	17 (7.4)	1.000
Postoperative procedures				
Percutaneous coronary intervention	15 (2.6)	13 (3.7)	2 (0.9)	.057
Cardiac surgery	129 (21.6)	62 (17.3)	67 (27.9)	.002
Abdominal surgery	27 (4.7)	16 (4.6)	11 (4.9)	1.000
Vascular surgery	67 (11.7)	43 (12.3)	24 (10.7)	.596
In-hospital mortality	372 (58.2)	206 (54.8)	166 (63.1)	.036
In-hospital mortality cause				.677
Multiorgan failure	121 (35.5)	68 (36.0)	53 (34.9)	
Sepsis	27 (7.9)	14 (7.4)	13 (8.6)	
Persistent heart failure	134 (39.3)	77 (40.7)	57 (37.5)	
Vasoplegia	5 (1.5)	2 (1.1)	3 (2.0)	
Bleeding	13 (3.8)	7 (3.7)	6 (3.9)	
Neurological injury	17 (5)	10 (5.3)	7 (4.6)	
Bowel ischemia	6 (1.8)	0 (0)	6 (3.9)	
Other	18 (5.3)	11 (5.8)	7 (4.6)	

Values are reported as n (% as valid percentage excluding missing values) or median (interquartile range). *CABG*, Coronary artery bypass graft; *LHV*, left heart valve.

### Comment

Data from the PELS analysis demonstrates that patients undergoing isolated CABG and those with concomitant LHV-CABG who require PC-ECLS exhibit distinct preoperative characteristics with a more severe risk profile in LHV-CABG patients. In almost half of LHV-CABG patients, the ECLS indication is failure to wean from CPB leading to a more frequent intraoperative ECLS initiation (65%), whilst ECLS implantation occurs postoperatively in 50% of isolated CABG cases. Finally, despite no major differences in early complications, LHV-CABG surgery carries an increased risk of in-hospital mortality compared with isolated CABG surgery. Nevertheless, we could not detect a difference in 5-year survival between groups when considering only patients who survived hospital discharge.

CABG combined with valve surgery carries higher operative risks compared with isolated CABG.^16^ Data from the current analysis underscore the influence of complex cardiac diseases, their consequent higher degree of preoperative organ dysfunction and surgical complexity on postcardiotomy cardiocirculatory compromise, and the need for ECLS and patient outcomes. As for other conditions,^17^, ^18^, ^19^ a preoperative patient's profile is intrinsically bound to the type of surgery and its consequences. Despite the fact that Cox models did not identify specific preoperative variables associated with in-hospital mortality besides age^20^ and surgical indication, patients should be considered in their complexity to implement a more preemptive and prophylactic approach to surgery. Patients undergoing LHV-CABG are older, 40.7% of them are women, and more frequently experience preoperative atrial fibrillation, pulmonary artery hypertension, right ventricular failure, septic shock, and active endocarditis, leading to a higher European System for Cardiac Operative Risk Evaluation II score. This profile suggests that the valvular indication is probably the leading indication for surgery, and the need for a coronary intervention is consequent to a concomitant finding or an intraoperative complication. The definition of such preoperative risk profile in valve/CABG patients should warn toward a more accurate preoperative patient's optimization in light of the concept of Type I protected cardiac surgery.^8^

As previously demonstrated,^18^ clinicians tend to initiate ECLS intraoperatively in more complex valve patients, favoring an earlier approach in cases of difficult CPB weaning as occurred in LHV-CABG patients in the current study. Such an approach might mitigate the negative effects of riskier operations when the need for ECLS is timely recognized.^8^ Despite this approach being more frequent in the LHV-CABG group, mortality remained higher in this population and further dedicated studies should investigate whether a preplanned Type II protected cardiac surgery approach could mitigate such negative outcomes in patients undergoing multiple surgical procedures.^8^

The same concept could be extended to patients undergoing isolated CABG with the typical profile of an unstable coronary disease presenting with recent myocardial infarction, preoperative cardiogenic shock and need for preoperative intra-aortic balloon pump and/or emergency surgery that we observed in this study. Despite this, 50% of patients undergoing isolated CABG received postoperative ECLS initiation, suggesting that these patients might experience sudden or rapidly evolving worsening conditions (ie, new perioperative myocardial infarction) requiring close monitoring and prompt implementation of hemodynamic support measures.

Prevention of complications has also been advocated as a possible tool to reduce postoperative mortality.^11^ Comparison between patients undergoing isolated CABG and LHV-CABG did not show differences in complications, except for higher blood loss in the first 24 hours that did not translate into higher rates of postoperative reoperations or increased transfusion requirements, pacemaker implantation, and reinterventions for cardiac reasons. Whether these factors were also determinants for postoperative mortality could not be demonstrated with our data. Indeed, after adjustment for covariates, age and concomitant LHV surgery were the primary variables associated with in-hospital mortality. Patients who underwent concomitant LHV-CABG showed an observed 8% higher in-hospital mortality and a 1.37 adjusted HR (95% CI, 1.02-1.84) for the same outcome. Such results support the need for a thorough preoperative evaluation and optimization of this specific patients’ group. Risk assessment should be performed timely, and patients and families should be informed about the higher risk of mortality in case of PC-ECLS after concomitant LHV-CABG operations.

Although in-hospital postoperative course and mortality limit the success of PC-ECLS, hospital survivors demonstrate good 5-year survival regardless of the type of surgery, which is consistent with previous studies on similar patients who did not receive postoperative ECLS.^21^, ^22^, ^23^ The favorable 5-year survival likely reflects that, once patients recover from the early postoperative and ECLS period, outcomes are no longer driven by the initial hit of complex surgery but instead by broader cardiac and noncardiac conditions. This highlights the importance of long-term follow-up and quality of life assessments among survivors.^24^^,^^25^

The observational design of this study precludes the establishment of causal relationships and it is inherently susceptible to confounding by indication. Nevertheless, a prevalent observational descriptive statistical approach was employed to represent the observed clinical reality. A potential selection bias cannot be ruled out because patient analysis was based on ECLS implantation following index surgery. This bias could arise from factors such as incomplete coronary revascularization, unplanned CABG or LHV surgery, or the decision not to proceed with a planned LHV surgery. Furthermore, potential center-specific surgical volumes and time-specific management variability,^26^ and use of hybrid approaches or interventional strategies to treat coronary or aortic valve diseases were not captured by the current database. Nevertheless, center and year effects were considered in all Cox models to mitigate effects of such variability. Given the timespan of inclusion, many patients were operated before the widespread adoption of microaxial transaortic flow pumps, transcatheter valve interventions, and during a time with more limited use of biological prostheses. The PELS database did not include details regarding the type of mitral or aortic valve disease (degenerative vs ischemic, regurgitation vs stenosis) precluding the investigation of this variable on outcomes of patients undergoing combined LHV-CABG. Additionally, data regarding the degree of frailty in this population was not collected.

## Conclusions

Despite the different preoperative profiles and no main difference in early complications, concomitant LHV-CABG surgery carries an intrinsically higher risk for in-hospital mortality compared with isolated CABG in PC-ECLS settings. Strategies to improve these outcomes should include a multidisciplinary heart team evaluation for potential transcatheter alternatives, preoperative risk stratification, early identification of patients undergoing LHV-CABG and likely to fail weaning from CPB, as well as timely ECLS initiation. Further investigations should focus on the role of a prophylactic ECLS approach in high-risk LHV-CABG operations, as well as on the identification of the best mechanical circulatory support for this type of patient.

## Conflict of Interest Statement

Prof Lorusso is a consultant for Medtronic-Abiomed-LivaNova, speaker for Abiomed, and an advisory board member of Eurosets-Hemocue-Xenios (honoraria as research funding). Prof Wiedemann is a consultant/proctor for Abbott and a scientific advisor for Xenios. Dr Ramanathan receives honorarium from Baxter and Fresenius for educational lectures and is chair of the ELSO Publications Committee. All other authors reported no conflicts of interest.

The *Journal* policy requires editors and reviewers to disclose conflicts of interest and to decline handling or reviewing manuscripts for which they may have a conflict of interest. The editors and reviewers of this article have no conflicts of interest.

## Figures and Tables

**Table E1:** Complete and missing cases for each study variable

Variables	Complete cases	Missing cases
Year	639 (100)	0 (0)
Age (y)	638 (99.8)	1 (0.2)
Sex	639 (100)	0 (0)
Body mass index	636 (99.5)	3 (0.5)
Comorbidities		
Hypertension	627 (98.1)	12 (1.9)
Dialysis	614 (96.1)	25 (3.9)
Previous acute myocardial infarction	639 (100)	0 (0)
Acute myocardial infarction (last 30 d)	627 (98.1)	12 (1.9)
Smoking	557 (87.2)	82 (12.8)
Previous stroke	639 (100)	0 (0)
Atrial fibrillation	638 (99.8)	1 (0.2)
Diabetes mellitus	639 (100)	0 (0)
Implanted pacemaker	594 (93.0)	45 (7.0)
Implanted cardioverter-defibrillator	596 (93.3)	43 (6.7)
Previous percutaneous coronary intervention	635 (99.4)	4 (0.6)
Chronic obstructive pulmonary disease	614 (96.1)	25 (3.9)
Pulmonary hypertension (>50 mm Hg)	636 (99.5)	3 (0.5)
Previous cardiac surgery	639 (100)	0 (0)
Left ventricular ejection fraction (%)	608 (95.1)	31 (4.9)
European System for Cardiac Operative Risk Evaluation II score	439 (68.7)	200 (31.3)
Pre-operative condition		
New York Heart Association functional class	609 (95.3)	30 (4.7)
Pre-operative cardiogenic shock	628 (98.3)	11 (1.7)
Pre-operative intubation	638 (99.8)	1 (0.2)
Pre-operative cardiac arrest	631 (98.7)	8 (1.3)
Pre-operative septic shock	622 (97.3)	17 (2.7)
Pre-operative vasopressors	632 (98.9)	7 (1.1)
Pre-operative right ventricular failure	558 (87.3)	81 (12.7)
Emergency surgery	630 (98.6)	9 (1.4)
Urgent surgery	631 (98.7)	8 (1.3)
Preoperative creatinine (μmol/L)	599 (93.7)	40 (6.3)
Diagnosis		
Active endocarditis	688 (100)	0 (0)
Coronary artery disease	639 (100)	0 (0)
Aortic valve disease	639 (100)	0 (0)
Mitral valve disease	639 (100)	0 (0)
Weight of surgery	639 (100)	0 (0)
Arterial conduits	504 (78.8)	135 (21.1)
Aortic valve surgery	639 (100)	0 (0)
Mitral valve surgery	639 (100)	0 (0)
Cardiopulmonary bypass time (min)	547 (85.6)	92 (14.4)
Crossclamp time (min)	540 (84.5)	92 (14.4)
Intraoperative transfusions of any blood components	253 (39.6)	386 (60.4)
Intraoperative lactates (mmol/L)	256 (40.1)	383 (59.9)
Extracorporeal life support indication	626 (98.0)	13 (2.0)
Extracorporeal life support implant timing	639 (100)	0 (0)
Blood loss first 24 h extracorporeal life support	262 (41.0)	377 (59.0)
Postoperative packed red blood cells transfusion	305 (47.7)	334 (52.3)
Intra-aortic balloon pump	633 (99.1)	6 (0.9)
Intra-aortic balloon pump timing	177 (25.7)	511 (74.3)
Left ventricular unloading	518 (81.1)	121 (18.9)
Extracorporeal life support duration (d)	629 (98.4)	10 (1.6)
Extracorporeal life support duration (h)	629 (98.4)	10 (1.6)
Mechanical ventilation time	416 (65.1)	223 (34.9)
Intensive care unit stay (d)	612 (95.7)	27 (4.2)
Hospital stay (d)	609 (95.3)	30 (4.7)
Post-operative complications		
Post-operative bleeding	631 (98.7)	8 (1.3)
Requiring rethoracotomy	602 (94.2)	37 (5.8)
Cannulation site bleeding	630 (98.6)	9 (1.4)
Diffuse no-surgical related bleeding	594 (93)	45 (7.0)
Neurological complications		
Intracerebral bleeding	611 (95.6)	28 (4.4)
Brain edema	619 (96.9)	20 (3.1)
Seizure	618 (96.7)	21 (3.3)
Cerebral hemorrhage	613 (95.9)	26 (4.1)
Stroke	634 (99.2)	5 (0.8)
Arrhythmia	597 (93.4)	42 (6.6)
Leg ischemia	608 (95.1)	31 (4.9)
Cardiac arrest	598 (93.6)	41 (6.4)
Pacemaker implant	597 (93.4)	42 (6.6)
Bowel ischemia	597 (93.4)	42 (6.6)
Right ventricular failure	585 (91.5)	54 (8.5)
Acute Kidney Injury	593 (92.8)	46 (7.2)
Pneumonia	583 (91.2)	56 (8.8)
Septic shock	583 (91.2)	56 (8.8)
Distributive shock syndrome	582 (91.1)	57 (8.9)
Postoperative procedures		
Percutaneous coronary intervention	573 (89.6)	66 (10.3)
Cardiac surgery	598 (93.6)	41 (6.4)
Abdominal surgery	573 (89.6)	66 (10.3)
Vascular surgery	575 (90)	64 (10)
In-hospital mortality	639 (100)	0 (0)
Main cause of in-hospital mortality	341 (91.7)	31 (8.3)
Survival group	633 (99.1)	6 (0.9)

Values are presented as n (%).

**Table E2:** Cox proportional hazards for significant determinants of in-hospital mortality∗

		95% CI		
Variables	Hazard ratio	Lower limit	Upper limit	*P* value
Model 1: Crude model with a random intercept for hospital				
CABG + left heart valve (Reference: isolated CABG)	1.28	1.03	1.58	.023
Model 2: Model 1 + demographic data and preoperative variables				
CABG + left heart valve (reference: isolated CABG)	1.26	1.00	1.58	.052
Age	1.03	1.02	1.04	< .001
Sex (reference: male)	1.03	0.81	1.30	.829
Previous myocardial infarction	0.90	0.71	1.14	.367
Atrial fibrillation	0.86	0.64	1.15	.309
Diabetes mellitus	1.12	0.89	1.39	.332
COPD	1.29	0.93	1.81	.129
Implanted pacemaker	0.87	0.53	1.43	.581
Implantable cardioverter defibrillator	0.94	0.41	2.14	.887
Pulmonary artery hypertension (>50 mm Hg)	1.14	0.83	1.56	.432
Previous cardiac surgery	1.19	0.83	1.71	.334
Left ventricular ejection fraction	1.00	1.00	1.01	.259
Preoperative creatinine	1.00	1.00	1.00	.344
Active endocarditis	1.00	0.48	2.07	.989
Preoperative cardiogenic shock	1.30	0.97	1.72	.076
Preoperative septic shock	0.99	0.44	2.22	.979
Preoperative intra-aortic balloon pump implantation	1.08	0.80	1.46	.611
Emergency surgery	1.07	0.80	1.43	.637
Model 3: Model 2 + intraoperative and ECLS variables				
CABG + left heart valve (reference: isolated CABG)	1.39	1.04	1.86	.025
Age	1.03	1.02	1.04	< .001
Sex (reference: male)	1.01	0.80	1.29	.906
Previous myocardial infarction	0.86	0.67	1.10	.220
Atrial fibrillation	0.82	0.61	1.11	.204
Diabetes mellitus	1.11	0.88	1.40	.389
COPD	1.30	0.93	1.82	.128
Implanted pacemaker	0.97	0.58	1.61	.903
Implantable cardioverter defibrillator	0.89	0.39	2.03	.775
Pulmonary artery hypertension (>50 mm Hg)	1.12	0.81	1.55	.503
Previous cardiac surgery	1.20	0.83	1.72	.334
Left ventricular ejection fraction	1.00	1.00	1.01	.314
Preoperative creatinine	1.00	1.00	1.00	.418
Active endocarditis	0.92	0.43	1.96	.830
Preoperative cardiogenic shock	1.32	0.98	1.78	.066
Preperative septic shock	1.08	0.48	2.45	.854
Preoperative intra-aortic balloon pump implantation	1.07	0.78	1.46	.679
Emergency surgery	1.04	0.78	1.39	.794
Crossclamp time	1.00	1.00	1.00	.277
Cardiopulmonary bypass time Postoperative ECLS implant (reference: intra-operative implant)	1.20	0.89	1.61	.229
ECLS indication (reference: failure to wean)				
Arrhythmia	0.55	0.26	1.16	.115
Cardiac arrest	1.10	0.72	1.68	.645
Cardiogenic shock	1.04	0.72	1.51	.838
Right ventricle failure	0.64	0.37	1.09	.098
Respiratory failure	0.83	0.42	1.66	.602
Biventricular failure	0.94	0.54	1.63	.813
Other	1.06	0.37	3.04	.911
Cannulation approach (reference: only peripheral)				
Only central cannulation	1.35	0.98	1.87	.069
Mixed/switch cannulation	1.13	0.86	1.49	.394
Model 4: Model 3 + complications				
CABG + left heart valve (reference: isolated CABG)	1.37	1.02	1.84	.037
Age	1.03	1.02	1.04	< .001
Sex (reference: males)	1.03	0.81	1.32	.785
Previous myocardial infarction	0.83	0.65	1.06	.132
Atrial fibrillation	0.80	0.59	1.10	.171
Diabetes mellitus	1.09	0.86	1.38	.484
COPD	1.27	0.90	1.78	.167
Implanted pacemaker	1.14	0.69	1.90	.607
Implantable cardioverter defibrillator	0.86	0.36	2.03	.728
Pulmonary artery hypertension (>50 mm Hg)	1.00	0.71	1.41	.999
Previous cardiac surgery	1.15	0.79	1.65	.467
Left ventricular ejection fraction	1.00	1.00	1.01	.423
Pre-operative creatinine	1.00	1.00	1.00	.540
Active endocarditis	0.87	0.40	1.87	.716
Preoperative cardiogenic shock	1.30	0.96	1.76	.086
Preoperative septic shock	1.05	0.46	2.41	.911
Preoperative IABP implantation	1.10	0.80	1.51	.558
Emergency surgery	1.03	0.77	1.39	.822
Crossclamp time	1.00	1.00	1.00	.424
Postoperative ECLS implant (reference: intra-operative implant)	1.22	0.91	1.64	.175
ECLS indication (Reference: failure to wean)				
Arrhythmia	0.61	0.29	1.29	.195
Cardiac arrest	1.10	0.72	1.69	.654
Cardiogenic shock	1.02	0.70	1.47	.935
Right ventricle failure	0.50	0.29	0.86	.013
Respiratory failure	0.80	0.40	1.61	.533
Biventricular failure	0.83	0.47	1.47	.529
Other	1.19	0.41	3.40	.752
Cannulation approach (reference: only peripheral)				
Only central cannulation	1.47	1.06	2.05	.023
Mixed/switch cannulation	1.17	0.88	1.55	.279
Complications				
Cerebral hemorrhage	1.14	0.64	2.02	.660
Stroke	0.68	0.47	1.00	.049
Right ventricle failure	1.82	1.32	2.51	.000
Pacemaker implant	0.34	0.12	1.00	.051
Percutaneous coronary intervention	0.65	0.25	1.68	.363
Cardiac surgery reoperation	1.01	0.75	1.36	.954
Bleeding requiring rethoracotomy	0.95	0.75	1.21	.684

*CABG*, Coronary artery bypass graft; *COPD*, chronic obstructive pulmonary disease; *ECLS*, extracorporeal life support.

∗ Sensitivity analysis after excluding patients who received treatments before 2011.

**Table E3:** Baseline characteristics of the overall population operated between 2011 and 2020

Variables	Overall population (N = 489)	Isolated CABG (n = 293)	LVH-CABG (n = 196)	*P* value
Age (y)	67.0 (59.0-73.5)	66.0 (58.0-73.0)	70 (61.9-74.2)	.02
Sex				.005
Female	163 (33.3)	83 (28.3)	80 (40.8)	
Male	326 (66.7)	210 (71.7)	116 (59.2)	
Body mass index	26.8 (24.0-30.1)	26.3 (24.2-30.0)	26.2 (23.9-30.5)	.993
Comorbidities				
Hypertension	376 (78.7)	225 (78.1)	151 (79.5)	.734
Dialysis	47 (9.9)	27 (9.4)	20 (10.5)	.697
Previous AMI	245 (50.1)	169 (58.0)	76 (38.8)	<.001
AMI (within past 30 d)	117 (24.5)	83 (28.8)	34 (17.9)	.007
Smoking	139 (31.4)	94 (33.9)	45 (27.1)	.140
Previous stroke	56 (11.5)	28 (8.9)	30 (15.3)	.031
Atrial fibrillation	79 (16.2)	41 (14.0)	38 (19.5)	.032
Diabetes mellitus	188 (38.4)	120 (41.0)	68 (34.7)	.184
Implanted pacemaker	24 (5.3)	10 (3.6)	14 (8.0)	.052
Implantable cardioverter defibrillator	9 (2.0)	1 (0.4)	8 (4.5)	.003
Previous percutaneous coronary intervention	116 (23.9)	73 (25.0)	43 (22.2)	.515
Chronic obstructive pulmonary disease	56 (11.9)	38 (13.2)	18 (9.8)	.308
Pulmonary artery hypertension (>50 mm Hg)	71 (14.6)	29 (9.9)	42 (21.5)	<.001
Previous cardiac surgery	49 (10.0)	20 (6.8)	29 (14.8)	.005
Left ventricular ejection fraction (%)	40 (30-55)	40 (30-55)	45 (30-60)	.008
EuroSCORE II	6.3 (2.7-17.0)	5.0 (2.3-15.0)	8.0 (3.4-20.0)	.002
Preoperative condition				
NYHA functional class				.495
Class I	24 (5.1)	16 (5.7)	8 (4.3)	
Class II	100 (21.4)	60 (21.5)	40 (21.3)	
Class III	203 (43.5)	114 (41.0)	89 (47.3)	
Class IV	140 (30.0)	89 (32.0)	51 (27.1)	
Preoperative cardiogenic shock	130 (26.8)	89 (30.6)	41 (21.1)	.028
Preoperative intubation	69 (14.1)	42 (14.3)	27 (13.8)	.895
Preoperative cardiac arrest	54 (11.2)	34 (11.6)	20 (10.6)	.769
Preoperative septic shock	12 (2.5)	1 (1.0)	9 (4.8)	.015
Preoperative vasopressors	93 (19.1)	61 (21.0)	32 (16.5)	.241
Preoperative right ventricular failure	21 (4.7)	8 (2.9)	13 (7.7)	.023
Emergency surgery	148 (30.8)	106 (36.4)	42 (22.1)	.001
Urgent surgery	89 (18.5)	51 (17.5)	38 (20.0)	.549
Preoperative creatinine (μmol/L)	97.3 (79.2-127.1)	96.8 (77.0-122.5)	103.2 (81.6-140.5)	.029
Active endocarditis	15 (3.1)	0 (0)	15 (7.7)	N/A

Values are reported as n (%) as valid percentage excluding missing values or median (interquartile range). *AMI*, Acute myocardial infarction; *EuroSCORE*, European System for Cardiac Operative Risk Evaluation score; *NYHA*, New York Heart Association; *N/A*, not applicable.

**Table E4:** Procedural characteristics of the population undergoing operation between 2011 and 2020

Variables	Overall population (n = 489)	Isolated CABG (n = 293)	LVH-CABG (n = 196)	*P* value
Arterial conduits				.042
No arterial grafts	123 (29.9)	70 (26.7)	53 (35.6)	
1 arterial graft	223 (54.3)	143 (54.6)	80 (53.7)	
2 arterial grafts	65 (15.8)	49 (18.7)	16 (10.7)	
Aortic valve surgery			118 (60.2)	N/A
Aortic valve surgery type				
Aortic valve repair			19 (19.6)	
Biological prosthesis			67 (69.1)	
Mechanical prosthesis			11 (11.3)	
Mitral valve surgery			104 (53.1)	N/A
Mitral valve surgery type				
Mitral valve repair			40 (41.7)	
Biological prosthesis			34 (35.4)	
Mechanical prosthesis			22 (22.9)	
Cardiopulmonary bypass time (min)	169 (112-235)	132 (97-189)	212 (161-295)	.001
Crossclamp time (min)	83 (49-124)	57 (27-84)	122 (89-153)	<.001
Intraoperative transfusions of any blood components	214 (96.0)	136 (95.8)	7 8 (96.3)	1

Values are reported as n (% as valid percentage excluding missing values) or median (interquartile range). *CABG*, Coronary artery bypass graft; *LHV*, left heart valve; *N/A*, not applicable.

**Table E5:** Details on extracorporeal life support population undergoing operation between 2011 and 2020

Variables	Overall population (n = 489)	Isolated CABG (n = 293)	LVH-CABG (n = 196)	*P* value
ECLS indication				<.001
Failure to wean	158 (33.0)	72 (25.0)	86 (45.0)	
Arrhythmia	20 (4.2)	18 (6.3)	2 (1.0)	
Cardiac arrest	58 (12.1)	43 (14.9)	15 (7.9)	
Cardiogenic shock	174 (36.3)	123 (42.7)	51 (26.7)	
Pulmonary hemorrhage	1 (0.2)	0 (0)	1 (0.5)	
RV failure	27 (5.6)	13 (4.5)	14 (7.3)	
Respiratory failure	14 (2.9)	6 (2.1)	8 (4.2)	
Biventricular failure	19 (4.0)	11 (3.8)	8 (4.2)	
Other	8 (1.7)	2 (0.7)	6 (3.1)	
ECLS cannulation site				.101
Only central	73 (14.9)	37 (12.6)	36 (18.4)	
Only peripheral	218 (44.6)	142 (48.5)	76 (38.8)	
Mixed/switch	187 (38.2)	109 (37.2)	78 (39.8)	
Unknown	11 (2.2)	5 (1.7)	6 (3.1)	
Distal perfusion cannula	199 (75.1)	133 (76.4)	66 (72.5)	.485
ECLS implant timing				<.001
Intraoperative	262 (53.6)	138 (47.1)	124 (63.3)	
Postoperative	227 (46.4)	155 (52.9)	72 (36.7)	
Chest status				.988
Closed	201 (55.5)	125 (55.6)	76 (55.5)	
Open	161 (44.5)	100 (44.4)	61 (44.5)	
Blood loss first 24 h ECLS (mL)	830 (420-1600)	695 (380-1500)	900 (500-2020)	.020
Postoperative PRBCs (n)	12 (5-26)	11 (6-26)	13.5 (5-26)	.844
IABP	211 (43.7)	148 (51.4)	63 (32.3)	<.001
IABP implant timing				.404
Preoperative	69 (32.7)	97 (65.5)	45 (71.4)	
Intraoperative	142 (67.3)	97 (65.5)	45 (71.4)	
Left ventricular unloading	170 (42.1)	116 (49.6)	54 (31.8)	<.001
ECLS duration (h)	126.3 (67.5-216.0)	141.0 (71.0-222.7)	120.0 (59.0-197.0)	.171

Values are reported as n (% as valid percentage excluding missing values) or median (interquartile range). *CABG*, Coronary artery bypass graft; *ECLS*, extracorporeal life support; *IABP*, intra-aortic balloon pump; *LHV*, left-heart-valve; *PRBCs*, packed red blood cells.

**Table E6:** Postoperative outcomes of the population undergoing operation between 2011 and 2020

Variables	Overall population (n = 489)	Isolated CABG (n = 293)	LVH-CABG (n = 196)	*P* value
Mechanical ventilation time (h)	10.0 (4.0-16.0)	9.0 (4.0-15.0)	10 (5.0-117.0)	.321
Intensive care unit stay (d)	14.0 (7.0-25.0)	14.0 (8.0-25.0)	14.0 (6.0-26.0)	.920
Hospital stay (d)	20.0 (12.0-35.0)	20.0 (12.0-35.0)	20.5 (8.0-36.5)	.422
Postoperative complications				
Postoperative bleeding	251 (52.2)	145 (50.3)	106 (54.9)	.352
Bleeding requiring rethoracotomy	161 (35.1)	89 (32.1)	72 (39.6)	.110
Cannulation site bleeding	59 (12.3)	35 (12.2)	24 (12.4)	1
Diffuse no-surgical related bleeding	141 (23.7)	87 (24.4)	54 (22.8)	.552
Neurological complications	340 (85.2)	215 (83.3)	125 (88.7)	.184
Intracerebral bleeding	9 (1.9)	4 (1.4)	5 (2.7)	.328
Brain edema	14 (3.0)	8 (2.8)	6 (3.2)	1
Seizure	11 (2.3)	6 (2.1)	5 (2.7)	.759
Cerebral hemorrhage	14 (3.0)	6 (2.1)	8 (4.4)	.174
Stroke	51 (10.5)	32 (11.0)	19 (9.8)	.763
Arrhythmia	165 (35.9)	110 (39.0)	55 (31.1)	.090
Leg ischemia	47 (10.1)	29 (10.3)	18 (9.8)	1.000
Cardiac arrest	78 (17.0)	43 (15.2)	35 (19.9)	.203
Pacemaker implant	8 (1.7)	2 (0.7)	6 (3.4)	.060
Bowel ischemia	26 (5.7)	17 (6.0)	9 (5.1)	.687
Right ventricular failure	62 (13.9)	32 (11.6)	30 (17.6)	.090
Acute kidney injury	232 (51.1)	136 (48.7)	96 (54.9)	.211
Pneumonia	86 (19.3)	55 (19.9)	31 (18.3)	.712
Septic shock	57 (12.8)	34 (12.3)	23 (13.6)	.770
Distributive shock syndrome	38 (8.6)	22 (8.0)	16 (9.5)	.602
Postoperative procedures				
Percutaneous coronary intervention	14 (3.2)	12 (4.3)	2 (1.2)	.092
Cardiac surgery	111 (24.1)	58 (20.5)	53 (29.9)	.025
Abdominal surgery	21 (4.7)	12 (4.3)	9 (5.4)	.647
Vascular surgery	54 (12.1)	34 (12.2)	20 (12.0)	1
In-hospital mortality	278 (56.9)	155 (52.9)	123 (62.8)	.031
Main cause of in-hospital mortality				.352
Multiorgan failure	93 (37.2)	55 (39.3)	38 (34.5)	
Sepsis	18 (7.2)	10 (7.1)	8 (7.3)	
Persistent heart failure	98 (39.2)	54 (38.6)	44 (40.0)	
Vasoplegia	5 (2.0)	2 (1.4)	3 (2.7)	
Bleeding	9 (3.6)	4 (2.9)	5 (4.5)	
Neurological injury	12 (4.8)	7 (5.0)	5 (4.5)	
Bowel ischemia	4 (1.6)	0 (0)	4 (3.6)	
Other	11 (4.4)	8 (5.7)	3 (2.7)	

Values are presented as n (%) as valid percentage excluding missing values or median (interquartile range). *CABG*, Coronary artery bypass graft; *LHV*, left heart valve.

**Table E7:** Comparison of valve prostheses use before and after 2011

Variables	2000-2010 (n = 67)	2011-2020 (n = 196)	*P* value
Aortic valve surgery type			.044
Aortic valve repair	12 (34.3)	19 (19.6)	
Biological prosthesis	16 (45.7)	67 (69.1)	
Mechanical prosthesis	7 (20)	11 (11.3)	
Mitral valve surgery type			.050
Mitral valve repair	22 (66.7)	40 (41.7)	
Biological prosthesis	6 (18.2)	34 (35.4)	
Mechanical prosthesis	5 (15.2)	22 (22.9)	

Values are presented as n (%).

**Table:** 

	Item no.	Recommendation	Page no.
Title and abstract	1	(a) Indicate the study’s design with a commonly used term in the title or the abstract - Retrospective, multicentre, observational study-Abstract	1
		(b) Provide in the abstract an informative and balanced summary of what was done and what was found - Provided in Abstract	7
Introduction			
Background/rationale	2	Explain the scientific background and rationale for the investigation being reported - Included in Background	7
Objectives	3	State specific objectives, including any prespecified hypotheses - Included in Background	7
Methods			
Study design	4	Present key elements of study design early in the paper - Included in Methods	7-8
Setting	5	Describe the setting, locations, and relevant dates, including periods of recruitment, exposure, follow-up, and data collection - Included in Methods	7
Participants	6	*Cohort study*—Give the eligibility criteria, and the sources and methods of selection of participants. Describe methods of follow-up - Included in Methods *Case-control study*—Give the eligibility criteria, and the sources and methods of case ascertainment and control selection. Give the rationale for the choice of cases and controls *Cross-sectional study*—Give the eligibility criteria, and the sources and methods of selection of participants	8-9
		(b) *Cohort study*—For matched studies, give matching criteria and number of exposed and unexposed *Case-control study*—For matched studies, give matching criteria and the number of controls per case - Not applicable	n/a
Variables	7	Clearly define all outcomes, exposures, predictors, potential confounders, and effect modifiers. Give diagnostic criteria, if applicable - Included in Methods, and Supplemental Methods	8
Data sources/measurement	8∗	For each variable of interest, give sources of data and details of methods of assessment (measurement). Describe comparability of assessment methods if there is more than one group - Included in Methods, and Supplemental Methods	8
Bias	9	Describe any efforts to address potential sources of bias - Included in Limitations	9-13
Study size	10	Explain how the study size was arrived at - Included in Methods	8
Quantitative variables	11	Explain how quantitative variables were handled in the analyses. If applicable, describe which groupings were chosen and why	9
Statistical methods	12	a) Describe all statistical methods, including those used to control for confounding - Included in Methods	8-9
		b) Describe any methods used to examine subgroups and interactions - Included in Methods	8-9
		c) Explain how missing data were addressed - Included in Methods	9
		*Cohort study*—If applicable, explain how loss to follow-up was addressed - Included in Methods and Limitations *Case-control study*—If applicable, explain how matching of cases and controls was addressed *Cross-sectional study*—If applicable, describe analytical methods taking account of sampling strategy	8-9
		e) Describe any sensitivity analyses	10
Results			
Participants	13∗	(a) Report numbers of individuals at each stage of study—eg, numbers potentially eligible, examined for eligibility, confirmed eligible, included in the study, completing follow-up, and analysed - Included in Results	10
		(a) Give reasons for non-participation at each stage - Not applicable	n/a
		(a) Consider use of a flow diagram - Figure E1	Suppl.22
Descriptive data	14∗	(a) Give characteristics of study participants (eg, demographic, clinical, social) and information on exposures and potential confounders - Included in Results	10
		(a) Indicate number of participants with missing data for each variable of interest - Included in Supplemental Tables	Suppl-13
		(a) *Cohort study*—Summarise follow-up time (eg, average and total amount) - Included in Results	10
Outcome data	15∗	*Cohort study*—Report numbers of outcome events or summary measures over time - Figure 1	21
		*Case-control study—*Report numbers in each exposure category, or summary measures of exposure	
		*Cross-sectional study—*Report numbers of outcome events or summary measures	
Main results	16	(a) Give unadjusted estimates and, if applicable, confounder-adjusted estimates and their precision (eg, 95% confidence interval). Make clear which confounders were adjusted for and why they were included - Included in Results and Figure 2, Supplementary Tables	10, 21, Suppl.14-21
		(b) Report category boundaries when continuous variables were categorized - Included in Results and Table 1, Table 2, Table 3, Table 4	10,17-20
		(c) If relevant, consider translating estimates of relative risk into absolute risk for a meaningful time period	
Other analyses	17	Report other analyses done—eg, analyses of subgroups and interactions, and sensitivity analyses - Sensitivity analysis included in Supplementary Materials	Suppl 17
Discussion			
Key results	18	Summarise key results with reference to study objectives - Included in Discussion	11-14
Limitations	19	Discuss limitations of the study, taking into account sources of potential bias or imprecision. Discuss both direction and magnitude of any potential bias - Included in Limitations	12-14
Interpretation	20	Give a cautious overall interpretation of results considering objectives, limitations, multiplicity of analyses, results from similar studies, and other relevant evidence - Included in Discussion	11-14
Generalisability	21	Discuss the generalisability (external validity) of the study results - Included in Discussion	11-14
Other information			
Funding	22	Give the source of funding and the role of the funders for the present study and, if applicable, for the original study on which the present article is based - Included in Declarations	3

An Explanation and Elaboration article discusses each checklist item and gives methodological background and published examples of transparent reporting. The STROBE checklist is best used in conjunction with this article (freely available on the Web sites of PLoS Medicine at http://www.plosmedicine.org/, Annals of Internal Medicine at http://www.annals.org/, and Epidemiology at http://www.epidem.com/). Information on the STROBE Initiative is available at www.strobe-statement.org. *N/A*, Not applicable.

∗ Give information separately for cases and controls in case-control studies and, if applicable, for exposed and unexposed groups in cohort and cross-sectional studies.

## Appendix E1 List of Post-Cardiotomy Extracorporeal Life Support Investigators

**Samuel Heuts, MD, PhD** (Cardiovascular Research Institute Maastricht, Maastricht, the Netherlands; and Department of Cardiothoracic Surgery, Maastricht University Medical Centre, Maastricht, the Netherlands)

**Dominik Wiedeman, MD, PhD** (Department of Cardiac Surgery, Karl Landsteiner University, University Clinic St, Pölten, St. Pölten, Austria)

**Luca Conci, MD** (Division of Cardiac Surgery, Medical University of Vienna, Vienna, Austria)

**Jawad Khalil, MD** (Department of Cardiac Surgery, Leipzig Heart Center, Leipzig, Germany)

**Sven Lehmann, MD** (Department of Cardiac Surgery, Leipzig Heart Center, Leipzig, Germany)

**Jean-Francois Obadia, MD, PhD** (Division of Cardiothoracic and Vascular Surgery, Pontchaillou University Hospital, Rennes, France)

**Antonio Loforte, MD, PhD** (Division of Cardiac Surgery, IRCCS Azienda Ospedaliero-Universitaria di Bologna, Bologna, Italy; and University of Turin, Turin, Italy)

**Davide Pacini, MD, PhD** (Division of Cardiac Surgery, IRCCS Azienda Ospedaliero-Universitaria di Bologna, Bologna, Italy)

**Udo Boeken, MD, PhD** (Department of Cardiac Surgery, Medical Faculty, Heinrich Heine University, Duesseldorf, Germany)

**Robertas Samalavicius, MD, PhD** (II Department of Anesthesiology, Centre of Anesthesia, Intensive Care and Pain Management, Vilnius University Hospital Santariskiu Klinikos, Vilnius, Lithuania)

**Erwan Flecher, MD** (Division of Cardiothoracic and Vascular Surgery, Pontchaillou University Hospital, Rennes, France)

**Dinis Dos Reis Miranda, MD** (Department of Intensive Care Adults, Erasmus MC, Rotterdam, the Netherlands)

**Kogulan Sriranjan, MD** (Department of Intensive Care Medicine, Center of Applied Medical Research, St Vincent's Hospital, Darlinghurst, New South Wales, Australia)

**Daniel Herr, MD** (Departments of Medicine and Surgery, University of Maryland, Baltimore, Md)

**Marco Di Eusanio, MD, PhD** (Cardiovascular Department, Azienda Ospedaliero Universitaria delle Marche, Ospedali Riuniti 'Umberto I – G.M. Lancisi – G. Salesi' Università Politecnica delle Marche, Ancona, Italy)

**Igor Vendramin, MD** (Division of Cardiac Surgery, Cardiothoracic Department, University Hospital of Udine, Udine, Italy)

**Graeme MacLaren, MD, PhD** (Cardiothoracic Intensive Care Unit, Department of Cardiac, Thoracic and Vascular Surgery, National University Hospital, Singapore)

**Kollengode Ramanathan, MD** (Cardiothoracic Intensive Care Unit, Department of Cardiac, Thoracic and Vascular Surgery, National University Hospital, Singapore)

**Alessandro Costetti, MD** (Cardiac Surgery Unit, Cardiac Thoracic and Vascular Department, Niguarda Hospital, Milan, Italy)

**Chistof Schmid, MD** (Department of Cardiothoracic Surgery, University Medical Center Regensburg, Regensburg, Germany)

**Roberto Castillo, MD** (ECLS Unit, Departamento de Anestesia, Clínica Las Condes, Las Condes, Santiago, Chile)

**Tomas Grus, MD** (2nd Department of Cardiovascular Surgery, First Faculty of Medicine, Charles University and General University Hospital in Prague, Prague, Czech Republic)

**Marco Solinas, MD** (Ospedale del Cuore Fondazione Toscana "G. Monasterio", Massa, Italy)

## Appendix E2. Strengthening the Reporting of Observational Studies in Epidemiology Statement

Checklist of items that should be included in reports of observational studies.

## Appendix E3 Methods

### Data Collection

The following predefined groups of data were collected:

• Demographic data: sex, age, race.
• Patients characteristics: European System for Cardiac Operative Risk Evaluation score, length, weight, serum creatinine level, left ventricular ejection fraction, comorbidities (hypertension, chronic kidney disease requiring dialysis, previous myocardial infarction, previous endocarditis, smoking, previous stroke, atrial fibrillation, previous pulmonary embolism, diabetes mellitus, previous transient ischemic attack, implanted pacemaker, implanted implantable cardioverter defibrillator, previous percutaneous coronary intervention, chronic obstructive pulmonary disease, peripheral artery disease, chronic pulmonary embolism, asthma, pulmonary hypertension, previous cardiac surgery, implanted left ventricular assist device, and New York Heart Association functional class.
• Preoperative status: urgency of the procedure, weight of intervention, planned intervention, preoperative cardiogenic shock, preoperative intubation, preoperative cardiac arrest, preoperative septic shock, preoperative vasopressors, preoperative acute pulmonary edema, preoperative intra-aortic balloon pump, preoperative right ventricular failure, and preoperative biventricular failure.
• Diagnosis: coronary artery disease, aortic vessel disease, aortic valve disease, mitral valve disease, tricuspid valve disease, pulmonary valve disease, postacute myocardial infarction, ventricular septal rupture, free wall/papillary muscle rupture, graft failure, active endocarditis, atrial septal defect, post-left ventricular assist device right ventricular failure, and other diagnosis.
• Coronary surgery: arterial graft, number of distal arterial anastomoses, left internal thoracic artery, right internal thoracic artery, radial artery, gastro-epiploic artery, other arterial graft, venous graft, number of distal venous anastomoses, and other coronary surgery.
• Valve surgery: valve surgery, aortic valve surgery, aortic valve procedure, mitral valve surgery, mitral valve procedure, pulmonary valve surgery, pulmonary valve procedure, pulmonary valve implant, tricuspid valve surgery, and tricuspid valve procedure.
• Aortic surgery: approach to aortic surgery, aortic ascending surgery, aortic arch surgery, and descending aortic procedure.
• Other cardiac surgeries: cardiac assist device, heart transplantation, rhythm surgery, additional pacemaker/implantable cardioverter defibrillator procedure, ventricular septal defect closure, atrial septal defect closure, ventricular surgery, pericardiectomy, pulmonary embolectomy/endarterectomy, other cardiac surgery, and other cardiac surgery description.
• Extracorporeal circulation: extracorporeal circulation duration, crossclamp duration, circulation arrest, cardioplegia characteristics, off-pump conversion.
• Extracorporeal life support (ECLS) variables: ECLS indication, chest status, cannulation approach, use of left ventricular vent, ECLS duration (hours), configuration change, and ECLS monitoring.
• In-hospital outcomes: deceased in hospital, deceased timing, intensive care unit stay (days), hospital stay (days), in-hospital mortality, death timing, postoperative bleeding (requiring re-thoracotomy, cannulation site bleeding, diffuse no-surgical related bleeding), neurological complications (brain oedema, cerebral hemorrhage, seizure, stroke, and vasospasm), arrhythmia, leg ischemia, cardiac arrest, pacemaker implant, bowel ischemia, right ventricular failure, acute kidney injury, pneumonia, septic shock, distributive shock syndrome, acute respiratory distress syndrome, multiorgan failure, and embolism.
• Postoperative procedures: percutaneous coronary intervention, new cardiac surgery, abdominal surgery, and vascular surgery.
• Outcomes at follow-up: mortality status and follow-up time.

### Variable Definitions

The following definitions were used for the main study variables:

• Sex: our research and manuscript have been developed in accordance to the international World Health Organization definitions of sex and gender where *gender* describes those characteristics of women and men that are largely socially created (including concepts such as cisgender and transgender), whereas *sex* encompasses those that are biologically determined (https://www.who.int/genomics/gender/en/). The same distinction is mirrored by the definitions of gender and sex given by several other international institutions such as the World Health Organization Regional Office for Europe (https://www.euro.who.int/en/health-topics/health-determinants/gender/gender-definitions), the Office for National Statistics and United Kingdom government (https://www.ons.gov.uk/economy/environmentalaccounts/articles/whatisthedifferencebetweensexandgender/2019-02-21), or the Canadian Institutes of Health Research (https://cihr-irsc.gc.ca/e/48642.html). Despite these definitions, *sex* and *gender* are often mistakenly used interchangeably in scientific literature, health policy, and legislation. In our study, we defined our patients based on biologically determined sex (male/female) and we did not include a further analysis of gender identity.E1, E2, E3
• Hypertension: Systolic blood pressure >140 mm Hg or diastolic blood pressure >90 mm Hg,^E4^ or use of antihypertensive agents to maintain normal blood pressure.
• Smoking: active (smoking during the past 30 days) and more than 100 cigarettes during lifetime.
• COPD: Diagnosis of chronic obstructive pulmonary disease, any Gold classification^E5^
• Pulmonary hypertension: Systolic pulmonary artery pressure >50 mm Hg.
• European System for Cardiac Operative Risk Evaluation II score proposing a risk assessment of cardiac surgical procedures that incorporates patient age, sex, diabetic status, pulmonary disease, neurological function, renal function, presence of active endocarditis, pre-operative state, procedural urgency, and procedure type.^E6^
• New York Heart Association functional class: Functional class of dyspnea according to the classification as proposed by the New York Heart Association.
• Preoperative cardiogenic shock: Preoperative state with life-threatening hypotension despite rapidly escalating inotropic support. Critical organ hypoperfusion with worsening acidosis and/or lactate levels.^E7^
• Preoperative cardiac arrest: Preoperative cardiopulmonary resuscitation in the 24 hours before surgery.
• Preoperative septic shock: Septic patients with vasopressor requirement to maintain mean arterial pressure >65 mm Hg and serum lactate levels >2 mmol/L in the absence of hypovolemia.^E8^
• Preoperative right ventricular failure: Evidence of right-sided structural and/or functional abnormalities in combination with clinical symptoms and signs of right ventricular failure.^E9^
• Preoperative biventricular failure: Biventricular dysfunction accompanied by both signs and symptoms of right-sided and left-sided heart failure.^E10^
• Emergency surgery: Surgery before the beginning of the next working day after the decision to operate is made.^E6^
• Urgent surgery: Patients not electively admitted for operation but requiring surgery during the current admission without a possibility to be discharged before undergoing the definite procedure.^E6^
• Aortic vessel disease: Any disease of the ascending aorta. aortic arch or proximal descending aorta warranting surgical correction during the current procedure.
• Aortic valve disease: Any aortic valve disease, including (prosthetic) aortic valve stenosis, regurgitation, and endocarditis.
• Mitral valve disease: Any mitral valve disease, including (prosthetic) mitral valve stenosis, regurgitation, and endocarditis.
• Tricuspid valve disease: Any tricuspid valve disease, including (prosthetic) tricuspid valve stenosis, regurgitation, and endocarditis.
• Active endocarditis: Patients still on antibiotic treatment for endocarditis at the time of surgery.^E6^
• Rhythm surgery: Surgical (either epicardial or endoepicardial) ablation performed for atrial or ventricular arrhythmia.
• Failure to wean: Failure to wean from cardiopulmonary bypass despite preload optimization and completeness of surgery.
• Arrhythmia: Refractory ventricular arrhythmia with uncontrollable hemodynamic consequences.
• Cardiac arrest: Abrupt loss of heart function despite acute and simple interventions such as pacing and defibrillation.
• Cardiogenic shock: State of life-threatening hypotension despite rapidly escalating inotropic support, critical organ hypoperfusion, with worsening acidosis and/or lactate levels.^E7^
• Right ventricular failure: Evidence of right-sided structural and/or functional abnormalities in combination with clinical symptoms and signs of right ventricular failure.^E9^
• Respiratory failure: Reversible pulmonary disease which cannot anymore be managed by conventional mechanical ventilation, despite optimization of pharmacological interventions with or without prone positioning.
• Biventricular failure: Biventricular dysfunction accompanied by both signs and symptoms of right-sided and left-sided heart failure.^E10^
• Chest closed: Any cannulation condition in which the sternum is closed irrespective location of cannulas.
• Chest open: Any cannulation condition in which the sternum is left open irrespective of skin closure.
• Stroke: Neurological dysfunction caused by focal brain or retinal ischemia with clinical symptoms lasting more or less than 24 hours, with or without permanent disability.
• Transient ischemic attack: A brief episode of neurological dysfunction caused by focal brain or retinal ischemia with clinical symptoms lasting <1 hour, without evidence of acute brain infarction.^E11^
• Arrhythmia: Any atrial or ventricular arrhythmia lasting more than 30 seconds.
• Leg ischemia: Clinical signs of lower extremity ischemia requiring intervention (either by vascular surgery or cannula removal).
• Bowel ischemia: Intestinal ischemia with elevated lactate levels requiring abdominal surgical intervention.
• Acute kidney injury: Postoperative requirement for dialysis while not on dialysis before or duplication of preoperative creatinine levels (and absolute creatinine level >177 μmol/L).
• Pneumonia: Any (suspected) pulmonary infection treated with antibiotics.
• Septic shock: Sepsis with vasopressor requirement to maintain mean arterial pressure >65 mm Hg and serum lactate levels >2 mmol/L in the absence of hypovolemia.^E8^
• Distributive shock syndrome: mean arterial pressure <50 mm Hg with cardiac index >2.5 L/min/m^2^, right atrial pressure <5 mm Hg, left atrial pressure <10 mm Hg, and low systemic vascular resistance (<800 dyne/s/cm^−5^) during intravenous norepinephrine infusion (>0.5 μg/kg/min).^E12^
• Multiorgan failure: Hypometabolic state with involvement of more than one organ as established by biochemical and/or radiological analysis.

### Statistical Analysis

Data were merged and analyzed using SPSS 26.0 (IBM Corp), and R version 4.1.2 (R Foundation for Statistical Computing). The full cohort was categorized into 2 study groups for comparison. Missing data analysis was conducted with the *mice: Multivariate Imputation by Chained Equations* R package.^E13^ The percentage of missing values was calculated for each variable (Table E1). Missing data patterns were investigated and were identified as missing completely at random.

Descriptive statistics were conducted on available data only and no imputations were performed for this purpose. Normality was investigated with Kolmogorov-Smirnov, Shapiro-Wilk, and inspection of histograms and Q-Q plots as appropriate. Homogeneity of variance was tested with the Levene's test. Demographic and clinical variables were expressed as numbers (percent) for categorical variables. For each 2 × 2 table, χ^2^ test was used if all expected cell counts were ≥5, otherwise Fisher exact test was used. Fisher-Freeman-Halton exact test for tables with more than 2 × 2 cells were used. Continuous variables that complied with normality were reported as mean (SD). If equal variances could be assumed, they were tested by independent Student *t* test, otherwise the Welch test was used. If they did not comply with normality, they were reported as median (interquartile range) and tested with a Mann-Whitney *U* test. We described the population characteristics and preoperative variables, intraoperative variables, variables while on ECLS and postoperative complications.

Survival was investigated with the Kaplan-Meier method and comparisons were performed with the Log-rank test (*survival* and *survminer* R packages). Patients’ loss to follow-up were included in survival analyses and were considered censored at the time of their last control. Curves were truncated when the number of patients at risk from the study groups dropped below 10% of the initial sample.

To estimate the associations between type of surgery and in-hospital mortality, we conducted a mixed-effects Cox proportional hazards regression, using the *Coxme: Mixed Effects Cox Models* R package. The random effect was used to account for the dependency of observations due to clustering in centers and years. Based on clinical practice and literature, we first estimated a crude model, which was subsequently adjusted for sets of variables deemed potential confounders for the association with mortality. The mixed-effects Cox proportional hazards and logistic regression models were developed on 5 imputed datasets after the imputation of variables with <20% missing data. Variables with more missing data were omitted from the models. We used the *mice: Multivariate Imputation by Chained Equations* R package for the imputation process. Five imputed datasets were created with “cart” method. Predictors included variables selected to be part of the mixed-Cox models that were run on each of these datasets, and results were pooled (*junkka/ehahelper: Helper Functions for Event History Analysis* R package) to obtain estimates as hazard ratios with their 95% CIs and *P* values. Based on the possible variations in ECLS management and prosthetic valve availability over the study period (Figure E2), a sensitivity analysis was performed after exclusion of patients who received a postcardiotomy ECLS before 2011. Direct comparison of valve prosthesis types between patients operated before and after 2011 was performed to test changes in surgical practices. A two-sided p-value of < 0.05 was considered statistically significant.
